# Exploring biomarkers and prognostic factors in uterine carcinosarcoma: An insight into L1CAM, CDX2, p53, and MSI status

**DOI:** 10.1371/journal.pone.0285447

**Published:** 2023-05-18

**Authors:** Jesse Lopes da Silva, Lucas Zanetti de Albuquerque, Fabiana Resende Rodrigues, Nina Carrossini Bastos, Isabele Avila Small, Elisa Bouret Campos Barroso, Fernando Lopes Cordero, Daniel de Souza Fernandes, Eduardo Paulino, Andreia Cristina de Melo

**Affiliations:** 1 Division of Clinical Research and Technological Development, Brazilian National Cancer Institute, Rio de Janeiro, Brazil; 2 Division of Pathology, Brazilian National Cancer Institute, Rio de Janeiro, Brazil; 3 Clinical Oncology Section, Brazilian National Cancer Institute, Rio de Janeiro, Brazil; 4 Gynecologic Oncology Section, Brazilian National Cancer Institute, Rio de Janeiro, Brazil; Sapporo Ika Daigaku, JAPAN

## Abstract

**Background:**

Uterine Carcinosarcomas (UCS) are a rare type of cancer composed of an admixture of high-grade carcinomatous and sarcomatous elements. Clinicopathological prognostic factors in UCS are well established, but studies that approach the impact of biomarkers in this unusual disease are scarce. The study objective was to evaluate the prevalence and prognostic impact of a panel of prominent biomarkers in uterine carcinosarcoma (UCS) using an immunohistochemical characterization with four biomarkers.

**Methods and findings:**

The internal database of a single Brazilian institution was carefully explored to select women diagnosed with UCS who were submitted to surgery and postoperative chemotherapy with carboplatin and paclitaxel between January 2012 and December 2017. Tissue microarrays containing UCS samples were evaluated by immunohistochemistry for L1CAM, CDX2, p53 and microsatellite instability markers. A total of 57 cases were included. The mean age was 65.3 years (standard deviation, SD 7.0). L1CAM was negative (score 0, no staining) in 27 (47.4%) patients. Of L1CAM-positive, 10 (17.5%) showed weak (score 1, <10%), 6 (10.5%) showed moderate (score 2, between 10–50%), and 14 (24.6%) showed strong L1CAM staining (score 3, ≧50%). dMMR occurred in 3 (5.3%) cases. The p53 was aberrantly expressed in 15 (26.3%) tumors. CDX2 was positive in 3 (5.3%) patients. The three-year progression-free survival (PFS) rate in the general population of the study was 21.2% (95% CI: 11.7–38.1) and the three-year overall survival (OS) rate was 29.4% (95% CI: 18.1–47.6). By multivariate analysis, the presence of metastases and CDX2-positive were significantly associated with poorer PFS (p < 0.001 and p = 0.002, respectively) and OS (p < 0.001 and p = 0.009, respectively).

**Conclusion:**

The strong influence of CDX2 on prognosis requires further investigation. Biological or molecular variability may have impaired the assessment of the impact of the other markers on survival.

## Introduction

Uterine Carcinosarcomas (UCS) are a rare type of gynecological tumors that represent less than 5% of endometrial cancers, usually affecting postmenopausal patients and presenting with aggressive behavior [1]. Unlike endometrioid tumors, 45% of cases are diagnosed as FIGO stage III or IV [2, 3]. They are composed of an admixture of high-grade carcinomatous and sarcomatous elements [4, 5]. It is now accepted that UCS are a subtype of epithelial endometrial carcinomas, where the sarcoma is derived from the carcinoma as a result of transdifferentiation during tumor evolution in a process called epithelial-mesenchymal transition (EMT) [6]. Together with the fact that most metastases contain the epithelial counterpart (as high as 90%) [7], platinum doublets are the regimen of choice for adjuvant and palliative settings. Known prognostic factors in UCS include clinical stage, tumors bigger than 5 cm, deep myometrial invasion, lymphovascular space invasion and sarcoma predominance [2, 7–9].

Initially identified in neuronal cells involved in axon outgrowth and guidance during central nervous system development, the L1-cell adhesion molecule (L1CAM) is a transmembrane molecule of 200- to 220-kDa critical for embryonic development [10]. Some of the main pathways responsible for the EMT process involve the downregulation of E-Cadherin and hormonal receptors and the upregulation of L1CAM [11]. This adhesion molecule is highly expressed in ovarian and endometrial carcinomas and correlates with advanced-stage disease, metastasis and shorter survival [12]. In vitro studies suggest that the cleavage and release of soluble particles of L1CAM in serum may participate in the processes of migration, invasion and escape from programmed cell death [13, 14].

The Caudal-related homeobox transcription factor 2 (CDX2) is frequently expressed in both neoplastic and non-neoplastic cells within the gastrointestinal system [15]. The diffuse and intense immunohistochemical expression of CDX2 is useful in distinguishing colorectal adenocarcinoma from other sites [16, 17]. Recent reports have also identified the presence of this marker in other sites, such as ovarian epithelial tumors (especially in mucinous tumors), pancreatic adenocarcinoma, and transitional cell bladder carcinoma [18–21]. However, some small reports have reported varying rates of CDX2 expression in endometrioid adenocarcinoma (1.4–27%) [20, 22–24]. While it is well-established that colorectal tumors with CDX2 expression have better overall and cancer-specific survival rates, the prognostic impact of CDX2 on gynecological tumors, including UCS, is underexplored [25].

By inactivating the p53 protein (p53), which is an essential factor for controlling cell growth, some mutations of the TP53 suppressor gene are likely to play a direct role in carcinogenesis [26]. Mutant p53, which has a longer half-life compared to the wild-type protein, can be detected through immunohistochemical assays and has been identified as a marker of poorer outcomes for endometrial tumors [27]. Nonetheless, data on p53 expression in carcinosarcoma is limited [28].

Microsatellite Instability (MSI) is a crucial pathway in the development of endometrial cancer and has emerged as a significant predictor of response to immunotherapy and survival in tumors from various sites, including endometrial adenocarcinomas [29–31]. The loss of expression of mismatch repair (MMR) enzymes MLH1, PMS2, MSH2, and MSH6, which can be detected using immunohistochemistry (IHC), may serve as a surrogate marker for expensive and inaccessible genetic testing, which remains the gold standard for detecting cases with high-MSI [29, 32]. However, the data on endometrial carcinosarcoma is still insufficient.

The identification of accessible biomarkers with clinical utility for the management of UCS still poses an unmet need. The current study aimed to provide a description of the prevalence and explore the prognostic value of L1CAM, CDX2, p53 and microsatellite instability status in patients with UCS.

## Materials & methods

### Ethics statement, study design, patient selection and data collection

This study has been approved by the Ethics in Human Research Committee of the Brazilian National Cancer Institute (INCA), Rio de Janeiro, Brazil, and was conducted following Good Clinical Practice guidelines [33]. As this is an observational retrospective study, the Institutional Review Board decided to waive the requirement for informed consent from all patients. The authors strictly followed the recommendations of the STrengthening the Reporting of OBservational studies in Epidemiology (STROBE) guidelines and were involved in the study design, writing, critical review and editing of the manuscript, and vouched for the accuracy and completeness of the reported data.

All women diagnosed with UCS who underwent surgery and received postoperative chemotherapy with the standard dose of carboplatin and paclitaxel every three weeks for six cycles [34] at INCA between January 2012 and December 2017 were identified through a meticulous search in the institutional database. Patients with inadequate or insufficient pathological samples and synchronous or anachronistic tumors were excluded from this cohort. Clinical data, including sociodemographic factors, staging, surgery, histological subtype (homologous versus heterologous), disease progression and survival were retrospectively obtained from medical records. The staging was performed based on the International Federation of Gynecology and Obstetrics (FIGO) criteria of 2009 [35].

### Immunohistochemistry

The tissue microarray (TMA) assembly was constructed from regions of the formalin-fixed paraffin-embedded surgical specimen of primary tumors with the highest tumor cellularity. The sample cores were punched at six 4-μm intervals, and sarcomatous and epithelial areas were well-represented. Negative controls were created by counterstaining with hematoxylin, while positive controls were used for comparison with the tumor cell staining. Additionally, the slides were evaluated based on the percentage of positive cells compared to the total number of cells.

The expression of L1CAM (Clone 14.10, Biolegend, diluted at 1:1000) in tumor cells was evaluated based on the degree of positivity, with a score of 0 indicating 0% of positivity, a score of 1 indicating 1–10%, a score of 2 indicating ≥10–50%, and a score of 3 indicating ≥50%. For survival analysis, L1CAM positivity was defined as ≥1% (score ≥1) of tumor cells showing membranous L1CAM staining [36]. CDX2 expression (clone EPR2764Y, Cell Marque, diluted at 1:700) was considered positive if the score was ≥1% [24]. Aberrant p53 expression (clone SP53, Cell Marque, diluted at 1:500) was defined as ≥80% of positive tumor cell nuclei [37]. Loss of expression was determined if there was no nuclear staining for any of MLH1 (clone G168-728, Cell Marque, diluted at 1:600), PMS2 (clone MRQ-28, Cell Marque, diluted at 1:700), MSH2 (clone G219-1129, Cell Marque, diluted at 1:1000), and MSH6 (clone 44, Cell Marque, diluted at 1:300). The IHC status was then classified into two groups: mismatch repair deficient (dMMR) if at least one marker lacked nuclear staining and mismatch repair proficient (pMMR) if all markers showed nuclear staining [38].

### Statistical analysis

Progression-free survival (PFS) was determined by measuring the time from the first administration of CP infusion until the earliest date of disease progression, recurrence, or death. Overall survival (OS) was measured from the initial CP infusion until death from any cause or until the patient’s status was censored if they were alive on the last day of data collection. The Kaplan-Meier method was utilized to estimate the median duration of PFS and OS. Patients were categorized based on an array of factors such as their age, body mass index (BMI), race, stage, omentectomy, residual disease, adjuvant radiotherapy, the existence of lymphovascular invasion (LVI), histological subtype, and the status of IHC markers. The Shapiro-Wilk test was used to evaluate the normality of continuous variables, while categorical variables were presented as absolute and relative frequencies.

To evaluate the association of IHC findings and clinicopathological features with staging, the Student’s t-test or χ2 test for p-values were employed. The Cox proportional hazards were used to calculate the crude Hazard Ratio (HR) for each variable. For multivariate analysis, all variables with an association with survival outcomes at a p-value < 0.30 were included, and the Akaike Information Criterion (AIC) was employed to select the most appropriate model for multiple Cox analysis. A p-value < 0.05 was considered statistically significant, and any missing data were excluded from the analysis. The R project version 3.5.3 was used to conduct the statistical analyses [39].

## Results

In this cohort, 57 women were examined. At the time of diagnosis, the average age was 65.3 years (range: 49 to 79 years). The majority of patients were non-Caucasian (42 cases, which accounts for 73.7% of the cohort), and most had advanced disease (42 cases, or 73.7%, had FIGO III/IV). Additionally, the heterologous subtype was present in 30 cases (52.6%) and positive LVI was found in 25 cases (43.9%).

Regarding the initial surgical approach, 37 patients (65%) underwent lymphadenectomy, 27 patients (47.4%) underwent omentectomy, and 39 patients (68.4%) had an optimal resection, which was defined as having a residual disease size of less than 1.0 cm. All patients received postoperative chemotherapy with carboplatin plus paclitaxel as per the inclusion criteria. Adjuvant radiotherapy was subsequently performed in 24 patients (42.8%). The clinicopathological characteristics are summarized in Table 1, while Table 2 provides information on the surgical and adjuvant approaches.

**Table 1:** Correlation of clinical-pathological characteristics and clinical stage.

Variables/Biomarkers	I e II (%)	III e IV (%)	Total (%)	Crude *p*-value
Number of patients (%)	15 (26.3)	42 (73.7)	57	-
Mean age, years (SD)	66.3 (8.4)	65.0 (6.5)	65.3 (7.0)	0.533
60-year-old cutoff				
< 60 years old	5 (33.3)	12 (28.6)	17 (29.8)	0.986
≥ 60 years old	10 (66.7)	30 (71.4)	40 (70.2)	-
Mean BMI kg/m^2^ (SD)	28.3 (7.3)	28.7 (6.3)	28.6 (6.5)	0.845
Race/Ethnicity				
Non-white	9 (60.0)	33 (78.6)	42 (73.7)	0.223
White	6 (40.0)	8 (19.0)	14 (24.6)	-
Missing	0 (0.0)	1 (2.4)	1 (1.8)	-
Histologic subtype				
Heterologous	7 (46.7)	23 (54.8)	30 (52.6)	0.781
Homologous	4 (26.7)	8 (19.0)	12 (21.1)	-
Missing	4 (26.7)	11 (26.2)	15 (26.3)	-
LVI				
Absent	6 (40.0)	10 (23.8)	16 (28.1)	0.234
Present	4 (26.7)	21 (50.0)	25 (43.9)	-
Missing	5 (33.3)	11 (26.2)	16 (28.1)	-
CDX2				
Negative	14 (93.3)	40 (95.2)	54 (94.7)	0.625
Positive	1 (6.7)	2 (4.8)	3 (5.3)	-
L1CAM				
0	6 (40.0)	21 (50.0)	27 (47.4)	0.746
1	2 (13.3)	8 (19.0)	10 (17.5)	-
2	2 (13.3)	4 (9.5)	6 (10.5)	-
3	5 (33.3)	9 (21.4)	14 (24.6)	-
L1CAM				
Negative	8 (53.3)	29 (69.0)	37 (64.9)	0.715
Positive	9 (46.7)	21 (31.0)	20 (36.1)	-
MMR				
dMMR	0 (0.0)	3 (7.1)	3 (5.3)	0.697
pMMR	15 (100.0)	39 (92.9)	54 (94.7)	-
p53_aberrant				
No	11 (73.3)	31 (73.8)	42 (73.7)	1.000
Yes	4 (26.7)	11 (26.2)	15 (26.3)	-

Abbreviations: SD, Standard deviation; BMI, Body mass index; LVI, Lymphovascular Invasion; L1CAM, L1-cell adhesion molecule; dMMR, deficient mismatch repair; pMMR, proficient mismatch repair; CDX2, Caudal-related homeobox transcription factor 2.

**Table 2:** Treatment data of the study population (N = 57).

Treatment	N (%)
Omentectomy	
Yes	27 (47.4%)
No	30 (52.6%)
Lymphadenectomy	
Yes	37 (65%)
No	20 (35%)
Adjuvant radiotherapy	
Yes	24 (42.8%)
No	32 (57.2%)
Residual disease	
R1/2	18 (31.6%)
R0	39 (68.4%)

R0, optimal tumor resection (defined as disease <1.0 cm); R1/2, residual tumor at primary cancer site or regional lymph nodes.

**Fig 1:**
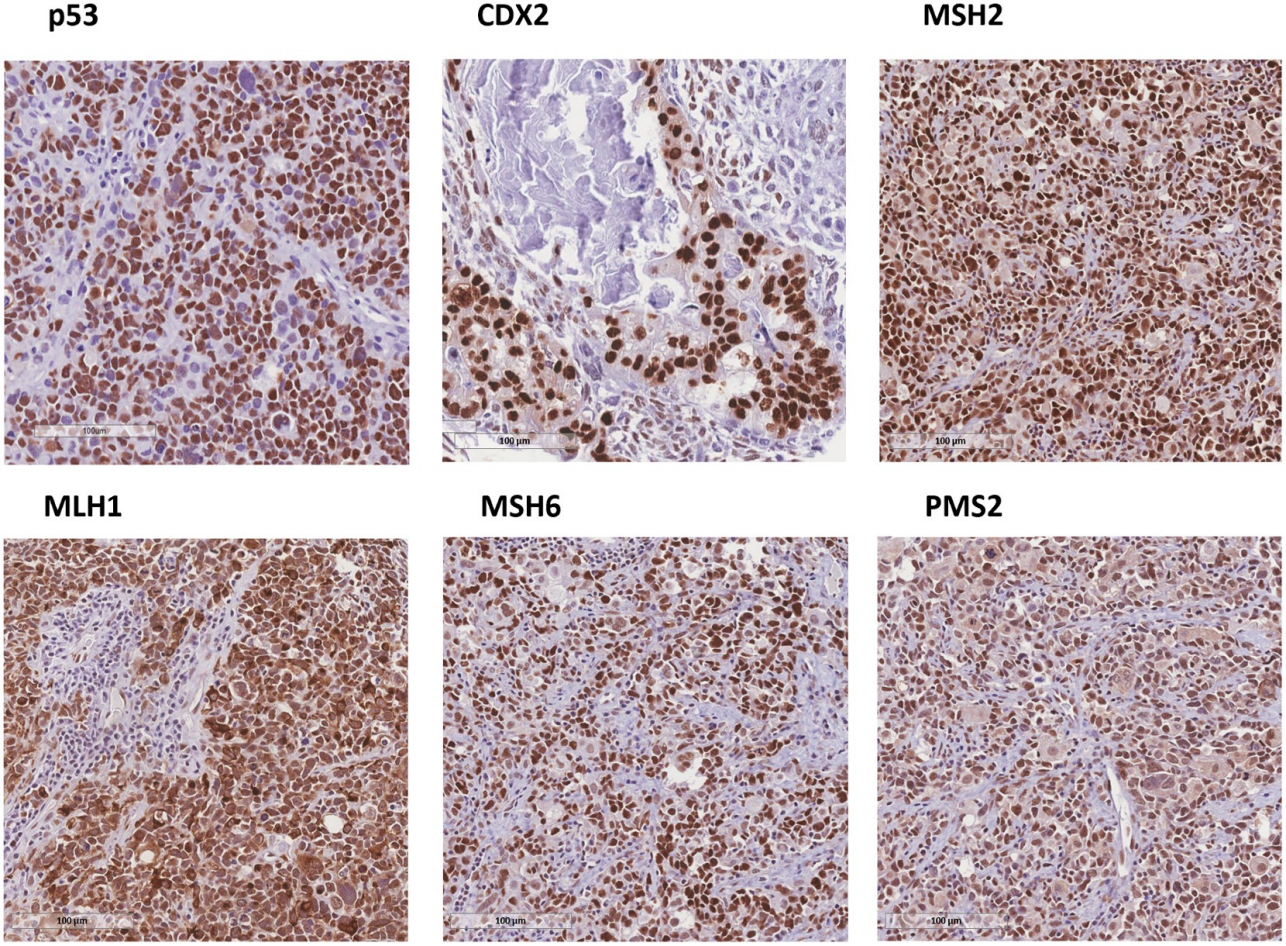
Immunohistochemistry biomarkers staining representative with high expression of p53, CDX2, MSH2, MLH1, MSH6 and PMS2. The visual data were captured at a magnification of x20 to ensure precision and are presented in this figure.

**Fig 2:**
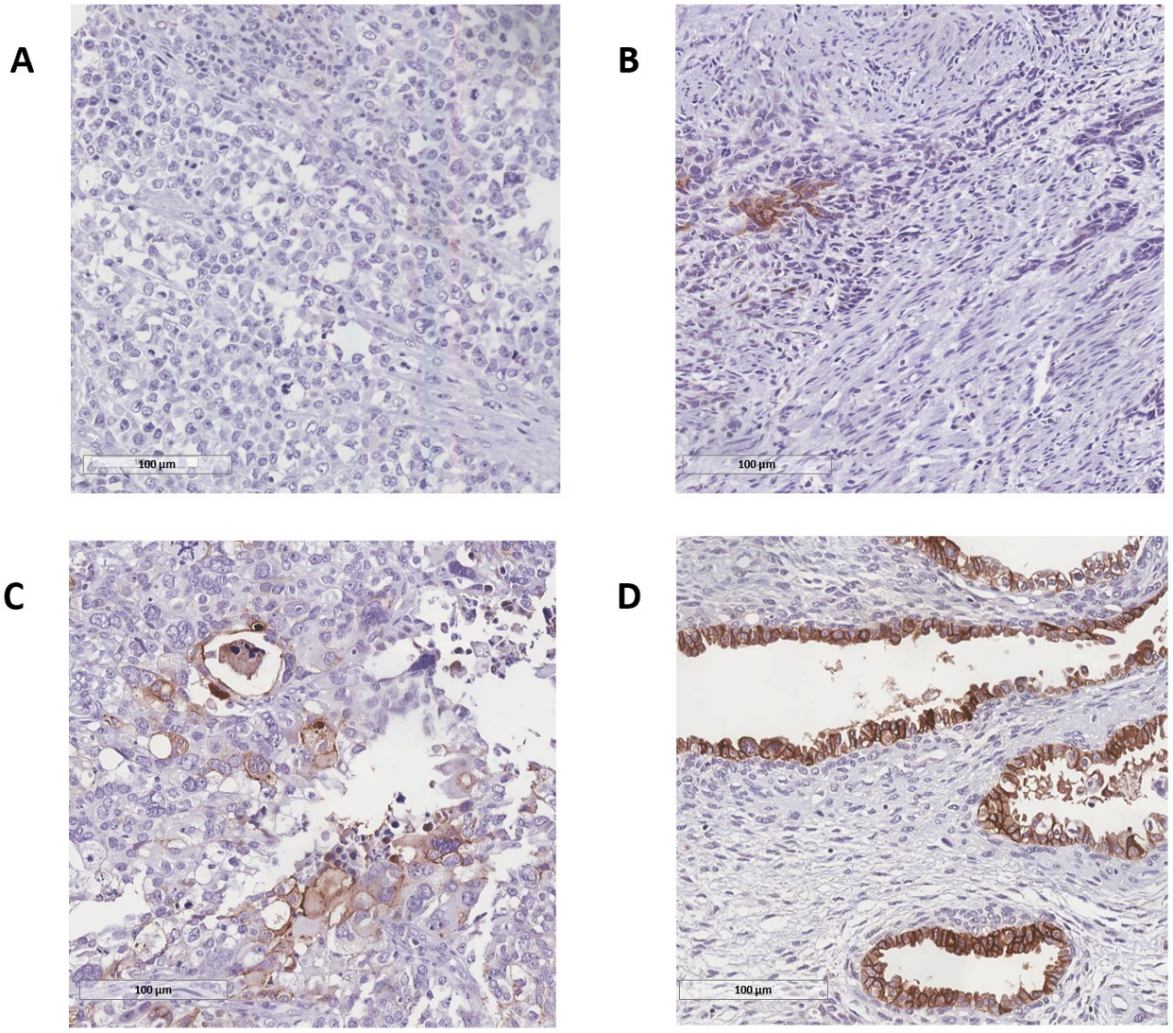
Scoring the L1CAM expression level in graduation ranks. The L1CAM expression level was assessed and scored as 0, 1+, 2+, and 3+ (A, B, C, and D, respectively) to quantify its intensity. The visual data were captured at a magnification of x20 to ensure precision and are presented in this figure.

As shown in Table 1, L1CAM was completely negative (score 0) in 27 (47.4%) patients. In L1CAM-positive tumors, 10 (17.5%) showed weak expression (score 1), 6 (10.5%) showed moderate (score 2), and 14 (24.6%) showed strong L1CAM staining (score 3). dMMR occurred in 3 (5.3%) cases. For p53, 15 (26.3%) tumors were aberrant. Finally, CDX2 was positive in 3 (5.3%) patients. There was no correlation between clinicopathological characteristics and IHQ expression. Fig 1 shows representative images of cases with high expression of p53, CDX2, MLH1, MSH2, MSH6 and PMS2 markers. Fig 2 displays the graduation score for L1CAM staining.

The median follow-up was 51 months (95% confidence interval, CI: 40–70), during which 42 patients experienced disease progression or died. The median PFS was 15 months (95% CI: 10–27), with a three-year PFS rate of 21.2% (95% CI: 11.7–38.1). Univariate analysis revealed that patients with FIGO stages III/IV (HR 2.7; 95% CI: 1.19–6.13; p = 0.017), metastasis (HR 3.28; 95% CI: 1.66–6.49; p = 0.001), residual disease (HR 4.19; 95% CI: 2.13–8.25; p < 0.001), and CDX2-positive tumors (HR 4.16; 95% CI: 1.21–14.32; p = 0.024) had a significantly higher risk of PFS events. For multivariate analysis of PFS, the Cox model included three variables: metastasis (present versus absent; HR 4.06; 95% CI: 1.98–8.34; p < 0.001), L1CAM (positive versus negative HR 0.65; 95% CI: 0.35–1.19; p = 0.162), and CDX2 (positive versus negative; HR 7.81; 95% CI: 2.10–28.99; p = 0.002). Table 3 provides a summary of the univariate and multivariate analyses for PFS.

**Table 3:** Crude and adjusted hazards ratios for carcinosarcoma progression-free survival (PFS) estimated by univariate analysis and multivariate analysis.

Clinicopathological features	Univariate analysis			Multivariate analysis		
	HR	95% CI	*p*-value	HR	95% CI	*p*-value
Mean age, years (SD)	1.03	0.99–1.07	0.171	-	-	-
Mean BMI kg/m^2^ (SD)	1.01	0.96–1.06	0.660	-	-	-
Race/Ethnicity (White vs non-white)	0.87	0.42–1.77	0.692	-	-	-
**Stage (III/IV vs I/II)**	**2.70**	**1.19–6.13**	**0.017**	**-**	**-**	**-**
**Metastasis (present vs absent)**	**3.28**	**1.66–6.49**	**0.001**	**4.06**	**1.98–8.34**	**<0.001**
Omentectomy (yes vs no)	0.61	0.33–1.14	0.120	-	-	-
**Residual disease (R1/2 vs R0)**	**4.19**	**2.13–8.25**	**<0.001**	**-**	**-**	**-**
Radiotherapy (yes vs no)	0.57	0.30–1.08	0.087	-	-	-
LVI (present vs absent)	1.06	0.50–2.25	0.872	-	-	-
Histological subtype (homologous vs heterologous)	0.83	0.39–1.78	0.631	-	-	-
p53_aberrant (present vs absent)	1.24	0.63–2.45	0.537	-	-	-
L1CAM (positive vs negative)	0.69	0.38–1.28	0.240	0.65	0.35–1.19	0.162
MMR (proficient vs deficient)	3.09	0.42–22.52	0.267	-	-	-
**CDX2 (positive vs negative)**	**4.16**	**1.21–14.32**	**0.024**	**7.81**	**2.10–28.99**	**0.002**

Abbreviations: BMI, Body mass index; L1cam, L1-cell adhesion molecule; LVI, Lymphovascular Invasion; MMR, mismatch repair; CDX2, Caudal-related homeobox transcription factor 2.

Note: Statistically significant results are in bold.

**Fig 3:**
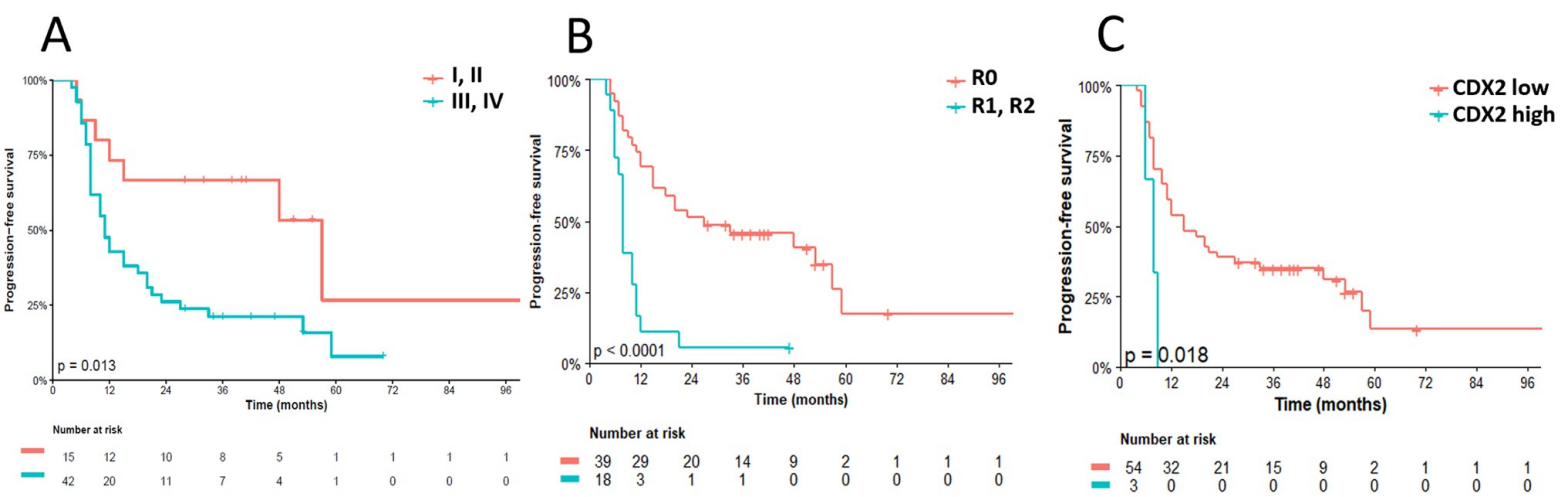
Progression-free survival (PFS). (A) stage status; (B) residual disease status (C) CDX2 status. Stratification of residual disease after surgery considered R0 when there was no evidence of residual disease and R1 or R2 correlate to microscopic and macroscopic residual disease, respectively. For immunohistochemistry biomarkers, Kaplan Meier curves for PFS were stratified by the median values as the cut-off for prognostic evaluation and divided into low vs high molecule expression patterns. The blue solid line indicates patients with high values and the red solid line low values. Tick marks indicate censored data.

**Fig 4:**
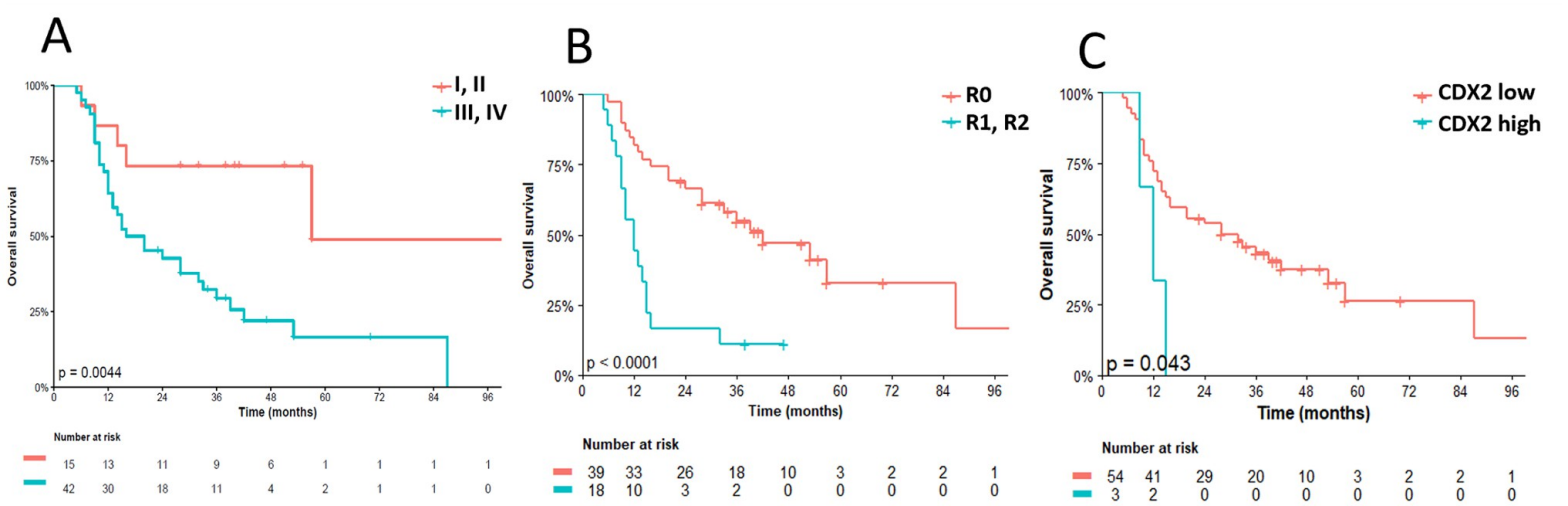
Overall survival (OS). (A) stage status (B) residual disease status (C) CDX2 status. Stratification of residual disease after surgery considered R0 when there was no evidence of residual disease and R1 or R2 correlate to microscopic and macroscopic residual disease, respectively. For immunohistochemistry biomarkers, Kaplan Meier curves for PFS were stratified by the median values as the cut-off for prognostic evaluation and divided into low vs high molecule expression patterns. The blue solid line indicates patients with high values and the red solid line low values. Tick marks indicate censored data.

At the time of analysis, 38 patients had died. The median OS was 28 months (95% CI: 15–53), with a three-year OS rate of 29.4% (95% CI: 18.1–47.6). Univariate analysis demonstrated that patients with FIGO stage III/IV (HR 3.62; 95% CI: 1.40–9.35; p = 0.008), metastasis (HR 3.23; 95% CI: 1.61–6.46; p = 0.001), residual disease (HR 3.83; 95% CI: 1.92–7.62); p < 0.001), and CDX2-positive tumors (HR 3.42; 95% CI: 1.01–11.60; p = 0.048) had a significantly higher risk of death. For multivariate analysis of OS, the Cox model also included three variables: metastasis (present versus absent; HR 3.93; 95% CI: 1.90–8.15; p < 0.001), L1CAM (positive versus negative; HR 0.57; 95% CI: 0.29–1.09; p = 0.091), and CDX2 (positive versus negative; HR 5.50; 95% CI: 1.54–19.69; p = 0.009). Table 4 provides a summary of the univariate and multivariate analyses for OS. Figs 3 and 4 depict the Kaplan-Meier curves for PFS and OS, respectively, according to the analyzed variables.

**Table 4:** Crude and adjusted hazards ratios for carcinosarcoma overall survival (OS) estimated by univariate analysis and multivariate analysis.

Clinicopathological features	Univariate analysis			Multivariate analysis		
	HR	95% CI	*p*-value	HR	95% CI	*p*-value
Mean age, years (SD)	1.02	0.98–1.07	0.307	-	-	-
Mean BMI kg/m^2^ (SD)	1.00	0.95–1.05	0.999	-	-	-
Race/Ethnicity (White vs non-white)	0.86	0.40–1.83	0.695	-	-	-
**Stage (III/IV vs I/II)**	**3.62**	**1.40–9.35**	**0.008**	-	-	-
**Metastasis (present vs absent)**	**3.23**	**1.61–6.46**	**0.001**	**3.93**	**1.90–8.15**	**<0.001**
Omentectomy (yes vs no)	0.63	0.33–1.23	0.175	-	-	-
**Residual disease (R12 vs R0)**	**3.83**	**1.92–7.62**	**<0.001**	-	-	-
Radiotherapy (yes vs no)	0.53	0.27–1.05	0.068	-	-	-
LVI (present vs absent)	1.05	0.47–2.35	0.900	-	-	-
Histological subtype (homologous vs heterologous)	0.84	0.38–1.87	0.671	-	-	-
p53_aberrant (present vs absent)	0.98	0.46–2.08	0.953	-	-	-
L1CAM (positive vs negative)	0.59	0.31–1.13	0.113	0.57	0.29–1.09	0.091
MMR (proficient vs deficient)	2.76	0.38–20.16	0.318	-	-	-
**CDX2 (positive vs negative)**	**3.42**	**1.01–11.60**	**0.048**	**5.50**	**1.54–19.69**	**0.009**

Abbreviations: BMI, Body mass index; L1cam, L1-cell adhesion molecule; LVI, Lymphovascular Invasion; MMR, mismatch repair; CDX2, Caudal-related homeobox transcription factor 2.

Note: Statistically significant results are in bold.

## Discussion

UCS is a rare disease, characterized by more advanced stages at diagnosis and poorer prognosis when compared to endometrioid carcinomas. The treatment generally consists of primary surgery followed by adjuvant chemoradiotherapy. In advanced disease, surgery can be considered the first approach when complete cytoreduction is deemed feasible. Regarding systemic therapy, carboplatin plus paclitaxel is the preferred regimen since it is likely to be non-inferior to paclitaxel and ifosfamide based on the results of the recently published GOG-0261 trial [40]. By the time the women included in the study were treated, platinum doublets had already been incorporated into the INCA routine as the standard of care for UCS.

The clinicopathological and sociodemographic characteristics observed in the current cohort are consistent with those reported by Matsuo et al. in a larger national database [1, 2, 41]. Of particular note are the higher prevalence of women over 60 years of age, obese individuals, and those with heterologous subtype tumors, as well as a significant presence of LVI. The proportion of cases diagnosed at advanced stages was considerable, with some patients having metastases detected only during or after surgery. The rates of omentectomy and lymphadenectomy in our cohort were also consistent with previously reported series [42–44].

As expected, the current analysis revealed a significant association between advanced tumor stage, metastasis, and residual disease with shorter PFS and OS. Possibly, the exclusion of staging and residual disease variables from the selected model based on the Akaike criteria was likely due to the strong correlation between these two variables impairing the statistical analysis, although no specific test was performed to confirm this hypothesis. Previous studies have already demonstrated the favorable impact of complete cytoreduction of UCS on OS. For instance, Tanner et al. reported a more favorable outcome in patients who achieved complete cytoreduction compared to those who underwent suboptimal surgery in a cohort of 44 patients with advanced carcinosarcoma (median OS of 52 versus 9 months, respectively, p < 0.0001) [45].

The mutational profile of UCS has not been thoroughly examined so far, unlike the endometrioid carcinoma setting. Although CDX2 was not previously linked to poorer outcomes in UCS, the current analysis reveals that it is infrequent in this population (5.3% of cases) but with a significant impact on prognosis. This finding may suggest that CDX2 is likely to be a marker of more aggressive behavior in UCS. CDX2 is highly expressed in various gastrointestinal cancers, such as colorectal adenocarcinomas (70–85%), gastroesophageal (50%), stomach (55–70%), ampulla (65–80%) and small intestine tumors (60%) [20, 46]. It can also be found in some non-gastrointestinal cancers, such as urachal, pulmonary mucinous, and certain gynecological tumors. In their study, Park et al. reported an incidence rate of 3.9% for malignant mixed Müllerian tumor cases [22]. In contrast, the other IHC markers (L1CAM, p53, and dMMR) used in this cohort did not significantly influence survival.

The Cancer Genome Atlas (TCGA) Research Network has identified four molecular subtypes of endometrioid and serous endometrial cancer based on the mutational burden, levels of copy number alterations, and MSI status. These subtypes include the POLE ultramutated group, the MSI hypermutated group, the copy number-high (serous-like) group, and the copy number-low (endometrioid) group, each of which has been associated with varying prognosis and progression-free survival outcomes [47–49]. In uterine carcinosarcomas (UCS), the majority of cases (60–78%) are classified as copy-number-high, with a smaller proportion of cases (22–38%) classified as copy-number-low. Only a minority of endometrial carcinosarcomas (< 5%) belong to the ultramutated or hypermutated groups [6, 47, 50]. The prevalence of p53 overexpression in UCS has been reported to range from 60–90% in previous studies, which is higher than the rates observed in the current cohort [6, 51–55]. However, methodological differences may explain this discrepancy. For both endometrioid and serous endometrial tumors, p53 overexpression is strongly associated with worse outcomes. In one study, Segura et al. found similar rates of p53 overexpression (20%) and dMMR (6.2%) in their cohort and described better outcomes for those UCS with dMMR compared to traditional pMMR [56]. In another study, Gotoh et al. found significant correlations between POLE, MSI, CNH, and CNL subtypes with patient outcomes in terms of progression-free survival and overall survival [6]. While the current study was not able to perform gene sequencing, the results for p53 and dMMR in this cohort were opposite to those reported by Gotoh et al. and Segura et al.

In lower-risk histologies, specifically endometrioid tumors, L1CAM has impactful prognostic value [10, 36, 57, 58]. The expression of p53 and L1CAM is positively correlated, with around 60–70% of p53 aberrant tumors also exhibiting L1CAM positivity (10% or higher) [10, 36, 57, 58]. However, in this population, L1CAM has not been shown to provide additional prognostic information [10, 36, 57, 58]. In the UCS scenario, the lack of prognostic impact of L1CAM may be explained by the increased expression of L1CAM that occurs only after the epithelial-mesenchymal transition (EMT) induction phase through exposure to the cytokine TGF-B1, which results in the upregulation of the EMT transcription factor Slug [58]. It is theoretically possible that cell migration and invasion would be more intensively triggered if this L1CAM upregulation process occurred at earlier stages [59]. The most significant contribution of L1CAM expression in tumor development may occur during the early stage of EMT, when cancer cells gain motility and invasive properties. This process could occur through direct action as an adhesion molecule or indirectly by acting as a cell-signaling molecule [11]. However, at a later stage of carcinogenesis, as may be the case for most patients in the present study, motility and invasive properties may have less impact on tumor progression and the metastasis process [60].

The strengths of this research rely primarily on the originality of the comprehensive evaluation of potentially valuable accessible biomarkers in the management of uterine UCS. The strict inclusion criteria ensured the selection of patients with homogeneous characteristics, favoring the assessment of the impact of clinicopathological features on disease progression and survival. The surgical samples were blindly examined by two experienced pathologists, and the results were consolidated using multivariate analyzes to enhance internal validity.

Conversely, the limitations of this study are closely related to its retrospective nature. In consequence, some confounding factors may be missing, which could weaken the quality of the analysis. Molecular analysis was not performed in this study, and opportunities to offer multimodal adjuvant treatment with chemotherapy followed by radiotherapy were decided by the care providers. Lastly, the small sample size may have been inadequate to ensure adequate statistical power to detect slight differences in survival for some IHC markers.

## Conclusion

Given the infrequency of UCS, conducting prospective randomized studies can be challenging. The current study assesses a panel of four biomarkers and presents some unprecedented findings in the field of UCS. The noteworthy impact of CDX2 on prognosis observed in this cohort warrants further investigation through multicenter studies with more extensive sample sizes. The survival outcomes were not influenced by the status of the other markers, implying the possibility of underlying biological or molecular variations.

## Supporting information

S1 Dataset (CSV)
